# COVID-19: we want all the brains back!

**DOI:** 10.1055/s-0043-1767765

**Published:** 2023-04-14

**Authors:** Caio César Diniz Disserol, Hélio Afonso Ghizoni Teive

**Affiliations:** 1 Universidade Federal do Paraná, Hospital de Clínicas, Departamento de Medicina Interna, Serviço de Neurologia, Unidade de Distúrbios do Movimento, Curitiba PR, Brazil.

Dear Editor,

The astonishing and encouraging paper by Yasuda in the issue of *Arquivos de Neuro-Psiquiatria* 1 highlights what all neurologists have encountered in the clinical practice since the beginning of the coronavirus disease 2019 (COVID-19) pandemic. The predicted “third wave” 2 seems to be an avalanche.

Symptoms that persist for several months can occur after an acute mild COVID-19 infection, what has been called “long COVID.” About one in four patients with long COVID has cognitive complaints. The first reports of these symptoms were found on social media, in which the term “brain fog” appeared. 3

We report here a similar interesting case of a 63-year-old woman. She is a schoolteacher focused on adult literacy, working in the field for many years, with an extensive vocabulary and high knowledge of grammar. A few weeks after a mild COVID-19 infection she had cognitive complaints that had never been present in her life. She reported inattention, forgetfulness, difficulty finding words, and problems in processing speed that led to an inability to handle complex tasks. She could no longer plan her classes due to executive dysfunction. The students complained of poor didacticism and missing necessary advice. Her performance was terrible, and she was temporarily removed from her job. Obviously, depressive symptoms developed. A long journey of rehabilitation was about to begin…

The patient began a treatment for depressive disorder, given that she was excluded from her job and social activities. With the improvement in the depressive symptoms with escitalopram, she was able to engage in multiple types of rehabilitation techniques: cognitive rehabilitation with a neuropsychologist and group activities, daily aerobic exercises, acupuncture sessions, guitar lessons, Pilates classes, and participation in social activities. After 1 year of these intensive nonpharmacological interventions, the patient could “clean” the brain fog, restore some cognitive performance, and was able to get back to work. Nevertheless, not yet at her baseline.

This common case encountered nowadays in outpatient clinics reflects the main cognitive domains affected by long COVID, especially executive function, as demonstrated by large cohorts. 4 Furthermore, it highlights the possible benefits of intensive nonpharmacological interventions for this condition. 5

Recently, new insights into the pathophysiology have been published. Glial dysregulation due to neuroinflammation, with a central role of the CCL11 cytokine, may cause neural circuit dysfunction and lead to brain fog. 2 Therefore, could its blockade be useful to treat brain fog? Additional research may answer this question.

We are grateful to you, Clarissa, for your courage to exemplify in a clear manner all those cognitive problems and show us that all we have recommended to our patients (intensification of exercises, medications, treating comorbidities) may not be enough. Unfortunately, until now, for most patients, the best remedy for this condition seems to be time. We hope that new evidence on the pathophysiology of brain fog gives rise to effective treatments.
